# Beneficial effects of magnesium nitrate on disease severity in male rats with monocrotaline‐induced pulmonary hypertension

**DOI:** 10.14814/phy2.70416

**Published:** 2025-06-17

**Authors:** Masashi Tawa, Shunto Abe, Ako Fujita, Riku Ando, Hajime Yoshinari, Keisuke Nakagawa, Mamoru Ohkita

**Affiliations:** ^1^ Department of Pathological and Molecular Pharmacology, Faculty of Pharmacy Osaka Medical and Pharmaceutical University Takatsuki Osaka Japan

**Keywords:** dietary nitrate, magnesium nitrate, monocrotaline, pulmonary hypertension, vasorelaxation

## Abstract

Inorganic nitrates and magnesium salts are effective in the treatment of pulmonary hypertension (PH). Here, we examined whether magnesium nitrate, which belongs to both categories, has a vasorelaxant effect on pulmonary arteries and protects against PH. In organ chamber experiments with isolated rat pulmonary arteries, high concentrations of magnesium nitrate (3 and 10 mM) produced weak relaxation, and phenylephrine‐induced contraction was attenuated in the presence of 3 mM magnesium nitrate. Exposure to magnesium nitrate (3 mM) did not affect acetylcholine‐induced relaxation. In rats with monocrotaline (MCT)‐induced PH, supplementation of drinking water with magnesium nitrate (3 and 10 mM) for 2 weeks from 2 weeks after MCT injection (60 mg/kg, s.c.) alleviated right ventricular systolic pressure elevation, regardless of the dose. High‐dose magnesium nitrate also suppressed pulmonary arterial medial thickening. Treatment with magnesium nitrate did not reduce right ventricular hypertrophy. Low‐dose magnesium nitrate tended to increase plasma nitrate levels, which increased significantly at a higher dose. High‐dose magnesium nitrate also increased plasma magnesium levels. These findings suggest that magnesium nitrate has antispasmodic effects on pulmonary arteries and is effective in halting the progression of PH, demonstrating its usefulness in managing PH.

## 1 INTRODUCTION

Pulmonary hypertension (PH) is a progressive disease with a poor prognosis and remains a highly unmet medical need (Mocumbi et al., 2024). Drugs that target the nitric oxide (NO)/soluble guanylate cyclase (sGC)/cGMP pathway, including sGC stimulators and phosphodiesterase type 5 inhibitors, are widely used for the pharmacological treatment of PH (Benza et al., 2024). Dietary inorganic nitrates have recently attracted attention as sources of NO that stimulate this pathway (Carlström et al., 2024). Nitrate (NO_3_^−^) can be converted to nitrite (NO_2_^−^) by bacteria in the oral cavity. Following this, some of the NO_2_^−^ is readily absorbed from the upper gastrointestinal tract into the circulation and reduced to NO by deoxyhemoglobin in the blood and xanthine oxidoreductase in various tissues (DeMartino et al., 2019). Deoxyhemoglobin and xanthine oxidoreductase are hypoxia‐inducible molecules; therefore, NO generation from NO_2_^−^ occurs preferentially under hypoxic conditions (DeMartino et al., 2019). Inorganic nitrate can therefore efficiently supply NO to hypoxic tissues such as the pulmonary circulatory system in PH. In this regard, accumulating evidence from animal studies shows that ingestion of inorganic nitrate, including potassium nitrate and sodium nitrate, inhibits PH‐associated pressure overload, pulmonary vascular remodeling, and right ventricular (RV) hypertrophy (Baliga et al., 2012; Bubb et al., 2014; Ogoshi et al., 2018; Tawa et al., 2021).

Magnesium (Mg^2+^) is an essential mineral with various physiological functions, including vasodilation (de Baaij et al., 2015). Specifically, Mg^2+^ induces direct vasodilation by acting as a natural calcium channel blocker and indirect vasodilation by stimulating the release of various vasoactive substances (Abidin et al., 2025). The pulmonary circulatory system is no exception, with the exposure of pulmonary arteries to high Mg^2+^ concentrations having been reported to attenuate vasoconstrictor‐induced contractions (Burke et al., 2011; Zhuang et al., 2022), evoke potent relaxation (Tolsa et al., 1999; Villamor et al., 1996), and potentiate agonist‐induced endothelium‐dependent relaxation (Fullerton et al., 1996; Zhuang et al., 2022). In PH, circulatory Mg^2+^ levels remain stable (Chaumais et al., 2012; Mathew et al., 1988; Wang et al., 2021); however, its levels in pulmonary arterial smooth muscle cells decrease (Wang et al., 2021; Xing et al., 2019). Several studies have revealed that Mg^2+^ salt supplementation is associated with low pressure in pulmonary circulation, mild pulmonary arterial wall thickening, and mild RV hypertrophy in a PH animal model (Chang et al., 2019; Mathew et al., 1988; Wang et al., 2021).

As mentioned above, both NO_3_^−^ and Mg^2+^ have protective effects on PH; therefore, their ionic compound, magnesium nitrate, may be an effective therapeutic candidate. This theory is supported by the fact that magnesium nitrate suppresses systemic blood pressure elevation in hypertensive conditions (Vilskersts et al., 2014). Additionally, it may have a therapeutic advantage over other inorganic nitrates, such as potassium nitrate and sodium nitrate. However, it remains unclear whether magnesium nitrate has a therapeutic effect on PH. We hypothesized that magnesium nitrate would have a stronger relaxant effect on pulmonary arteries than potassium nitrate or sodium nitrate and would inhibit the progression of PH. To test this hypothesis, we first examined the pulmonary vascular effects of magnesium nitrate ex vivo and then conducted an in vivo study using a rat model of monocrotaline (MCT)‐induced PH to elucidate its therapeutic effect.

## 2 MATERIALS AND METHODS

### 2.1 Animals

A total of 68 male Sprague–Dawley rats (8 weeks old, Japan SLC, Inc., Shizuoka, Japan) were used in this study. The rats were housed in an environmentally controlled room with a 12‐h light–dark cycle and had free access to adequate chow (NMF, Oriental Yeast Co., Ltd., Tokyo, Japan) and water. The Experimental Animal Committee at the Faculty of Pharmacy, Osaka Medical and Pharmaceutical University, approved the use of the animals (Permit no: AP23‐021), and all experiments were conducted in accordance with the Guide for the Care and Use of Laboratory Animals.

### 2.2 Organ bath experiments

The rats were anesthetized using sodium pentobarbital (40 mg/kg, i.p.; P0776, Tokyo Chemical Industry Co., Ltd., Tokyo, Japan) and meloxicam (1.0 mg/kg, s.c.; Inflacam® 0.5%, Virbac Japan Co., Ltd., Osaka, Japan). They were then injected with heparin (500 U/kg, i.v.; Heparin Sodium Injection 50,000 units/50 mL, NIPRO Co., Osaka, Japan) and sacrificed by bleeding from the abdominal aorta. The extralobar pulmonary arteries were isolated, the connective tissues were cleaned off, and the arteries were carefully dissected into 3–5‐mm rings with special care to preserve the endothelium. The rings were suspended horizontally between two stainless steel wires in an organ bath, with one wire connected to a fixed holder and the other connected to a force‐displacement transducer (TB‐611T, Nihon Kohden Kogyo Co., Tokyo, Japan). The bath was filled with Krebs–Ringer bicarbonate solution and gassed with carbogen (95% O_2_ and 5% CO_2_, 37°C) to maintain a pH of 7.4. Changes in isometric force were recorded using a PowerLab system (PL3508/P, ADInstruments, Castle Hill, Australia). The resting tension was adjusted to 0.7 g, and the preparations were allowed to equilibrate for 60–90 min. Thereafter, the viability of the preparation was determined with phenylephrine (PE, 1 μM; P0395, Tokyo Chemical Industry Co., Ltd.) followed by acetylcholine (ACh, 1–10 μM; 011‐00592, FUJIFILM WAKO Pure Chemical Co., Osaka, Japan). Viable preparations were repeatedly washed and allowed to return to the resting tension. Subsequently, preparations were challenged with a single dose of PE (1 μM), and a concentration‐response curve for magnesium nitrate (20919‐25, Nacalai Tesque, Kyoto, Japan), potassium nitrate (28704‐85, Nacalai Tesque), or sodium nitrate (31617‐35, Nacalai Tesque) was obtained. At the end of each experiment, papaverine (100 μM; Papaverine Hydrochloride Injection 40 mg/1 mL, Dainippon‐Sumitomo Pharma Co., Osaka, Japan) was added as a reference standard for maximal relaxation (100%).

In another experiment, the contractile response to 60 mM potassium chloride (KCl; 28513‐85, Nacalai Tesque) was measured twice, followed by repeated washouts with Krebs–Ringer bicarbonate solution. The second response was considered 100% contraction. After equilibration, the rings were exposed to each nitrate (3 mM) or the corresponding solvent in the bathing solution for 10 min. A concentration‐response curve for PE (1 nM–1 μM) was then obtained by adding the drug directly to the bathing solution in cumulative concentrations. Finally, a concentration‐related response to ACh (10 nM–100 μM) and a subsequent response to papaverine (100 μM) were observed.

### 2.3 Animal model

PH was induced in rats by MCT (C2401, Sigma‐Aldrich Co. LLC, St. Louis, MO, USA) injection (60 mg/kg, s.c.), as described previously (Tawa et al., 2022). The rats were grouped as follows: Control (*n* = 6, 0.9% saline injection + drinking water), MCT (*n* = 12, MCT injection + drinking water), MN‐L (*n* = 10, MCT injection +3 mM magnesium nitrate), and MN‐H (*n* = 9, MCT injection +10 mM magnesium nitrate). Supplementation with drinking water or magnesium nitrate‐dissolved drinking water was started 2 weeks (14 days) after saline or MCT injection and was maintained for an additional 2 weeks (14 days). The solutions were changed every 1–2 days. The average water intake per day was as follows: Control 47 mL, MCT 38 mL, MN‐L 40 mL (magnesium nitrate intake, 320 μmol/kg/day), and MN‐H 41 mL (magnesium nitrate intake, 1084 μmol/kg/day). Four weeks (28 days) after saline or MCT injection, hemodynamic measurements were performed. Following this, blood samples were collected from the inferior vena cava, and then the lungs and heart were harvested. Plasma, separated from blood by centrifugation at 3000 rpm for 10 min at 4°C, was stored at −80°C until use.

The effects of potassium nitrate and sodium nitrate were determined in the same manner as described above for magnesium nitrate. Briefly, the rats were grouped as follows: Control (*n* = 3), MCT (*n* = 6), PN (*n* = 7, MCT injection +10 mM potassium nitrate), and SN (n = 7, MCT injection +10 mM sodium nitrate). The average water intake per day was as follows: Control 45 mL, MCT 36 mL, PN 34 mL (potassium nitrate intake, 954 μmol/kg/day), and SN 36 mL (sodium nitrate intake, 980 μmol/kg/day).

### 2.4 Hemodynamics

Each rat was anesthetized as described above. During the experiment, body temperature was maintained using a heating pad (KN‐475, Natsume Seisakusho Co., Ltd., Tokyo, Japan). After stable anesthesia was achieved, the right carotid artery was isolated and incised, and a polyethylene catheter (SP‐31, Natsume Seisakusho Co., Ltd.) was inserted to record the arterial pressure and heart rate. A second catheter (PE 60, Becton Dickinson, Sparks, NV, USA) was advanced into the right ventricle through the right jugular vein to measure RV systolic pressure (RVSP). Blood pressure and heart rate signals were recorded on a personal computer via an analog‐to‐digital converter (PowerLab/4sp, ADInstruments).

### 2.5 Pulmonary vascular pathology

The left lung was placed in 10% phosphate‐buffered formalin, and the midsection of the lung was processed for microscopic examination by paraffin embedding. Four‐micrometer‐thick sections were stained using the Elastica van Gieson method (40322, 40342, 40352, 40362, and 40372, Muto Pure Chemicals Co., Ltd., Tokyo, Japan) to identify the pulmonary arteries with elastic laminae. The medial wall thickness was calculated using the following equation: ((external diameter minus internal diameter)/(external diameter)) × 100. At least 10–15 muscular arteries with an external diameter of 30–100 μm per lung section were examined using image viewer software (OlyVIA, Olympus). Values obtained were averaged for each animal and group.

### 2.6 Biochemical examination

Plasma NO_3_^−^ levels were measured using an ENO‐20 NOx Analyzer HPLC system (Eicom, Kyoto, Japan), as described previously (Tawa et al., 2022). The levels were calculated by comparison with values obtained from a standard solution (NO‐STD, Eicom).

Plasma Mg^2+^ levels were measured using a magnesium assay LS kit (MG01M, Metallogenics Co., Ltd., Chiba, Japan) according to the manufacturer's instructions. The levels were calculated by interpolating a standard curve generated using known concentrations.

### 2.7 Statistics

All values are expressed as the mean ± standard deviation (SD). Statistical analyses were performed using the GraphPad Prism 7.0 software (GraphPad Software Inc., San Diego, CA, USA). Concentration‐response curves were analyzed using two‐way repeated‐measures analysis of variance (ANOVA) and Holm–Sidak post hoc tests. All other data were compared using one‐way ANOVA, followed by the Holm–Sidak multiple comparison test. Statistical significance was set at *p* < 0.05.

## 3 RESULTS

### 3.1 Differences in pulmonary vascular effects among different inorganic nitrates

**FIGURE 1:**
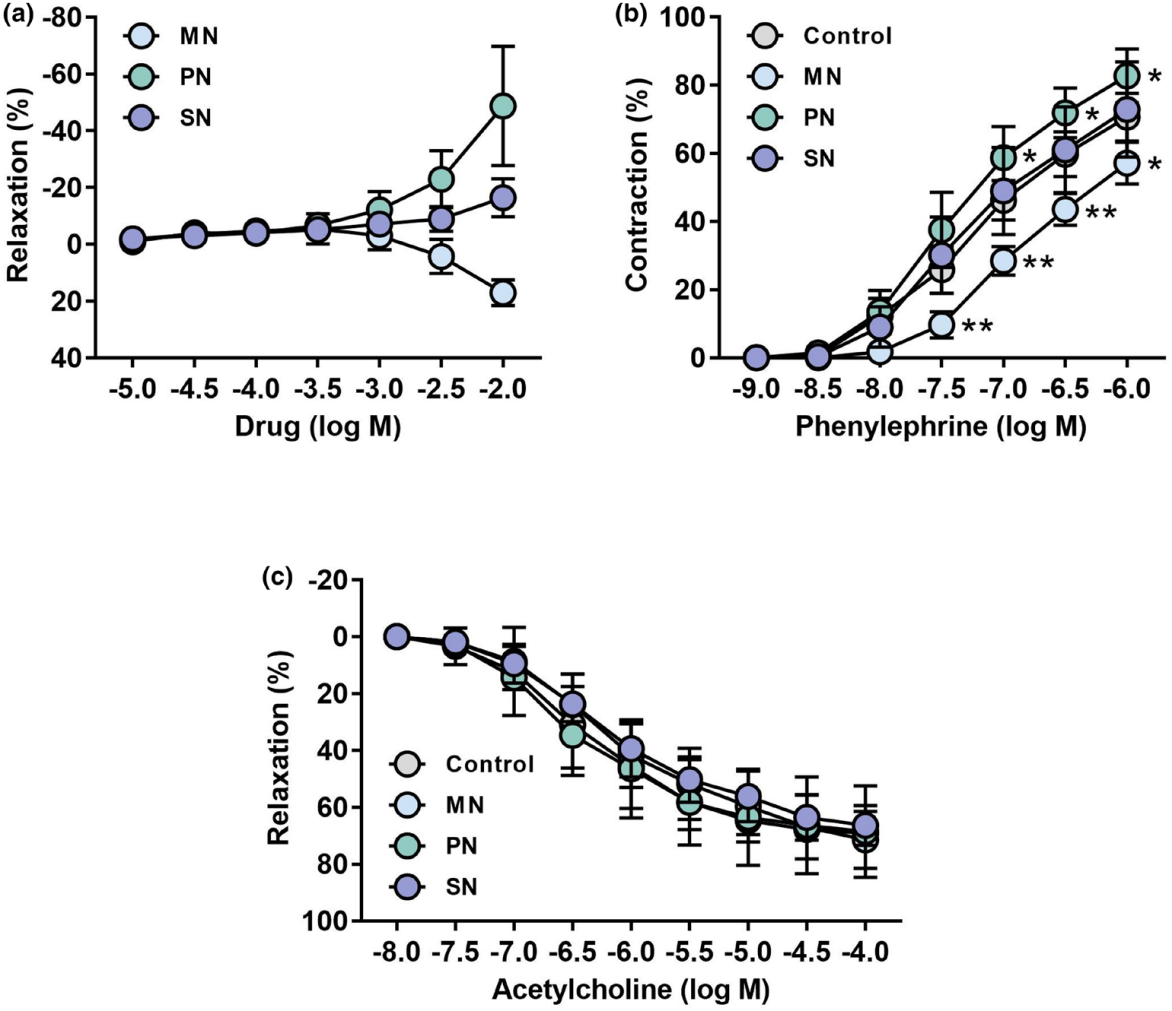
Vascular reactivity in isolated rat pulmonary arteries: (a) inorganic nitrate‐induced vasorelaxation; (b) phenylephrine‐induced vasocontraction in the presence or absence of an inorganic nitrate; (c) acetylcholine‐induced vasorelaxation in the presence or absence of an inorganic nitrate. Each point and bar represent the mean ± SD of four experiments. *p < 0.05 and **p < 0.01, compared with the Control. Statistical analysis was performed using two‐way repeated‐measures ANOVA and the Holm–Sidak post hoc test. MN, magnesium nitrate; PN, potassium nitrate; SN, sodium nitrate.

Magnesium nitrate did not produce any change in the tone of precontracted pulmonary arteries up to 1 mM, but showed a weak but relaxing effect at 3 and 10 mM. In contrast, high concentrations of potassium nitrate induced contractions rather than relaxation, whereas sodium nitrate did not induce relaxation (Figure 1a). The PE‐evoked concentration‐dependent contractions were significantly attenuated in the presence of magnesium nitrate. Potassium nitrate significantly augmented the response to PE, whereas sodium nitrate did not (Figure 1b). In the presence of either nitrate, the relaxant response to ACh did not differ from that of the control (Figure 1c).

### 3.2 Effects of magnesium nitrate on biometric parameters

The first sign of PH is weight loss due to lack of appetite. Body weight (g) in the MCT group (359.8 ± 23.8) was significantly (*p* < 0.001) lower than that in the Control group (436.3 ± 25.3); there was no difference in body weight between the MCT group and the MN‐L (371.2 ± 29.5) or MN‐H (372.8 ± 25.9) groups. As for central hemodynamics, heart rate (bpm) and mean arterial pressure (mmHg) did not differ among the groups (Control group, 380.9 ± 64.0 and 111.4 ± 17.0; MCT group, 381.4 ± 38.0 and 92.5 ± 17.6; MN‐L group, 400.0 ± 48.2 and 107.8 ± 25.6; MN‐H group, 378.3 ± 64.6 and 98.0 ± 28.1), indicating no systemic hemodynamic abnormalities.

### 3.3 Effects of magnesium nitrate on RV pressure elevation

**FIGURE 2:**
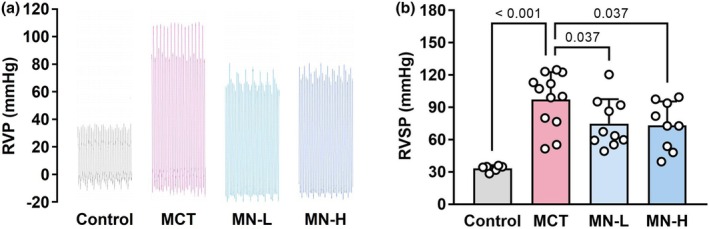
Right ventricular function in saline‐ and MCT‐injected rats; (a) typical records of RVP; (b) RVSP. Each point represents an individual value; each column and bar represent the mean ± SD of 6–12 experiments. Numbers above the line marks represent the calculated *p* value. Statistical analysis was performed using one‐way ANOVA and the Holm–Sidak post hoc test. MCT, monocrotaline; MN‐H, high‐dose magnesium nitrate; MN‐L, low‐dose magnesium nitrate; RVP, right ventricular pressure; RVSP, right ventricular systolic pressure.

RVSP, a surrogate marker of pulmonary artery systolic pressure, was three‐fold higher in the MCT than in the Control group (Figure 2a), indicating the presence of severe PH. There was a nonsignificant but attenuated elevation in RVSP in the MN‐L group and a significant attenuation of the elevation in the MN‐H group (Figure 2a,b).

### 3.4 Effects of magnesium nitrate on pulmonary arterial remodeling

**FIGURE 3:**
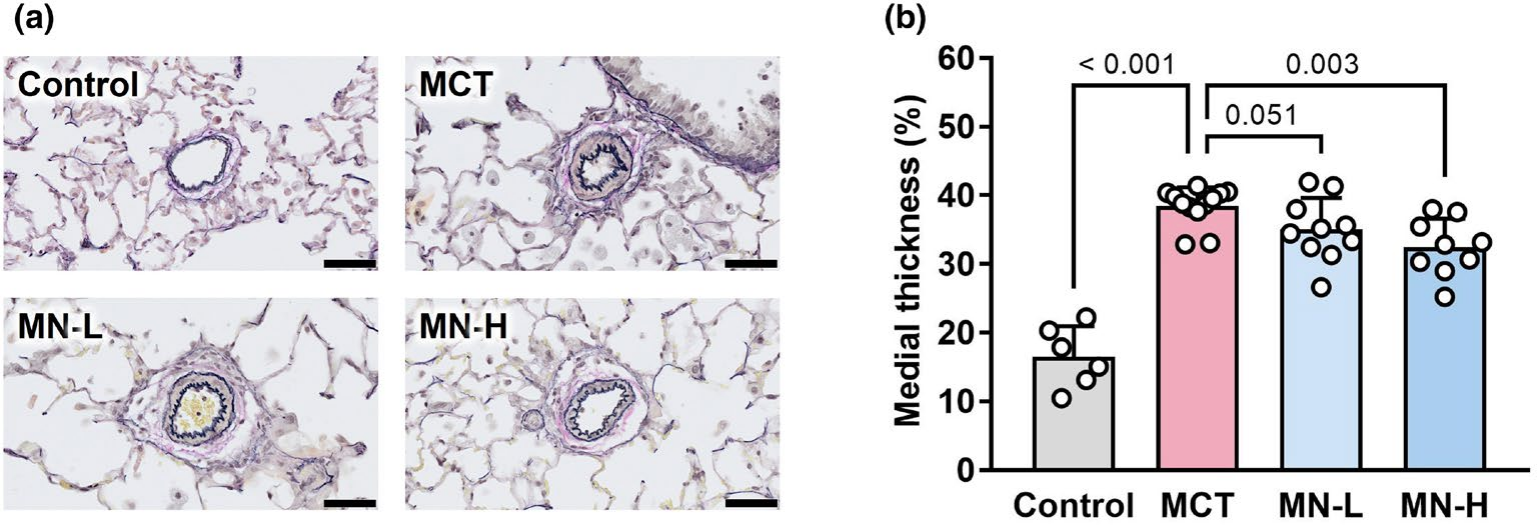
Pulmonary artery morphology in saline‐ and MCT‐injected rats; (a) typical images of a small pulmonary artery (scale bar, 50 μm); (b) medial thickness. Each point represents an individual value; each column and bar represent the mean ± SD of 6–12 experiments. Numbers above the line marks represent calculated *p* value. Statistical analysis was performed using one‐way ANOVA and the Holm–Sidak post hoc test. MCT, monocrotaline; MN‐H, high‐dose magnesium nitrate; MN‐L, low‐dose magnesium nitrate.

Figure 3a shows representative images of the small pulmonary arteries in the four experimental groups. Pulmonary arterial wall thickness was significantly increased in the MCT group compared to that in the Control group, indicating pulmonary vascular remodeling. Compared with the MCT group, wall thickness was reduced in the MN‐L group and significantly reduced in the MN‐H group (Figure 3b).

### 3.5 Effects of magnesium nitrate on RV hypertrophy

**FIGURE 4:**
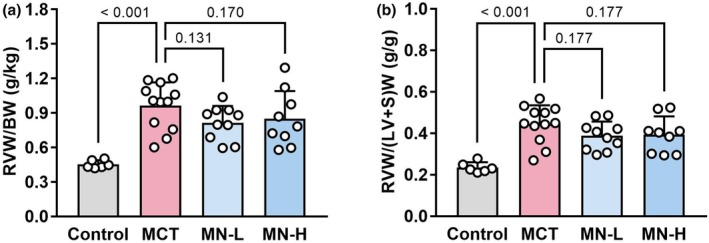
Right ventricular weight index in saline‐ and MCT‐injected rats; (a) RVW to BW ratio; (b) RVW to (LV + S)W ratio. Each point represents an individual value; each column and bar represent the mean ± SD of 6–12 experiments. Numbers above the line marks represent calculated *p* value. Statistical analysis was performed using one‐way ANOVA and the Holm–Sidak post hoc test. (LV + S)W, left ventricular plus septum weight; BW, body weight; MCT, monocrotaline; MN‐H, high‐dose magnesium nitrate; MN‐L, low‐dose magnesium nitrate; RVW, right ventricular weight.

The RV weight to body weight ratio and Fulton index, the RV weight to left ventricular plus septum (LV + S) weight ratio, were significantly increased in the MCT group compared to the Control group, indicating the existence of RV hypertrophy. None of these measured parameters were statistically different between the MCT and MN‐L or MN‐H groups, although there was a slight downward trend (Figure 4a,b).

### 3.6 Effects of magnesium nitrate on lung edema

Lung weight to body weight ratio (mg/g) in the MCT group (6.85 ± 0.93) was markedly (*p* < 0.001) higher than that in the Control group (3.41 ± 0.15), indicating the existence of lung edema. The values in the MN‐L (6.31 ± 0.90) and MN‐H (6.08 ± 1.20) groups were not significantly different from those in the MCT group.

### 3.7 Effects of magnesium nitrate on NO_3_ ^−^ and Mg^2+^ levels

**FIGURE 5:**
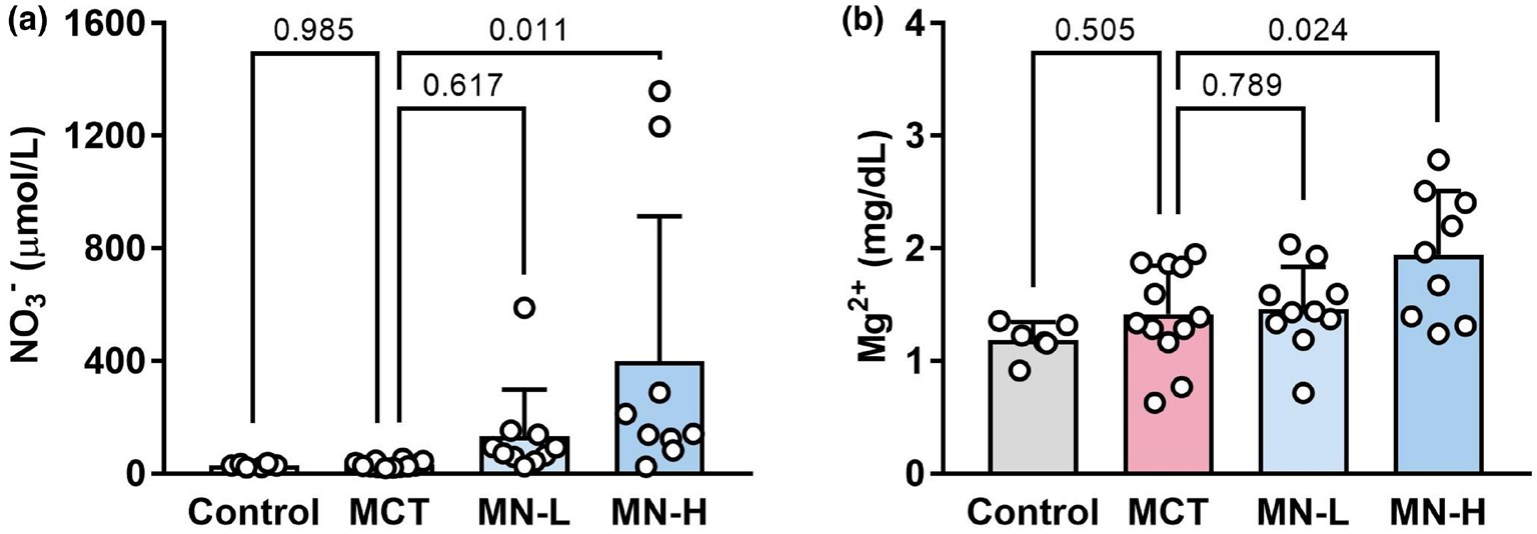
Plasma biochemical markers in saline‐ and MCT‐injected rats; (a) NO_3_^−^ levels; (b) Mg^2+^ levels. Each point represents an individual value; each column and bar represent the mean ± SD of 6–12 experiments. Numbers above the line marks represent calculated *p* value. Statistical analysis was performed using one‐way ANOVA and the Holm–Sidak post hoc test. MCT, monocrotaline; Mg^2+^, magnesium; MN‐H, high‐dose magnesium nitrate; MN‐L, low‐dose magnesium nitrate; NO_3_^−^, nitrate.

Plasma NO_3_^−^ levels did not differ between the Control and MCT groups. The levels were elevated in the MN‐L and MN‐H groups, with the latter showing a significant difference from the MCT group (Figure 5a). Similarly, the Mg^2+^ levels were significantly higher in the MN‐H group than in the MCT group (Figure 5b).

### 3.8 Effects of potassium nitrate and sodium nitrate on PH

Biometric parameters, including body weight, heart rate, mean arterial pressure, RV weight to body weight ratio, and lung weight to body weight ratio, did not differ between the MCT and PN or SN groups (Table S1). RVSP, pulmonary arterial wall thickness, and Fulton index, which are indicators of PH, did not differ between the groups (Figure S1a–c). Plasma NO_3_^−^ levels in the PN and SN groups were higher than those in the MCT group, although the difference was not statistically significant (Figure S1d).

## 4 DISCUSSION

In the present study, we established the pulmonary vascular effects of magnesium nitrate for the first time. Magnesium nitrate evoked the relaxation of PE‐contracted preparations and attenuated PE‐induced contraction, whereas other nitrates did not. In line with our results, sodium nitrate has been shown to induce minimal relaxation in rat pulmonary vascular beds contracted with U‐46619 (Nossaman et al., 2004). Potassium nitrate has been reported to cause cell swelling‐induced vasocontraction through elevation of extracellular potassium (K^+^) in rat aortas (Suzuki et al., 1981). In addition, slight extracellular K^+^ levels have been shown to enhance PE‐induced vasocontraction (Fransen et al., 2015). In pulmonary arteries, it is well known that Mg^2+^ has an inhibitory action on vasocontraction (Burke et al., 2011; Zhuang et al., 2022) and a vasorelaxant activity (Tolsa et al., 1999; Villamor et al., 1996); therefore, the antispasmodic effects of magnesium nitrate may be attributed to Mg^2+^ rather than NO_3_^−^.

Magnesium nitrate did not affect ACh‐induced endothelium‐dependent relaxation, which was also true for potassium nitrate and sodium nitrate. Regarding the effect of Mg^2+^ on pulmonary artery endothelial function, Mu et al. reported a slight enhancement in ACh‐induced relaxation in mouse pulmonary arteries when extracellular Mg^2+^ was 3.6 mM above the physiological concentration (Zhuang et al., 2022). In contrast, Oberleithner et al. showed that similar conditions produced a very slight attenuation of ACh‐induced relaxation in piglet pulmonary arteries (Villamor et al., 1996). The effect of high Mg^2+^ levels on pulmonary artery endothelial function may not be significant. However, none of the inorganic nitrates improved or impaired pulmonary artery endothelial function. Taken together, magnesium nitrate appears to be more effective than potassium nitrate and sodium nitrate in preventing excessive pulmonary vasoconstriction.

MCT‐induced PH is characterized by pulmonary artery endothelial cell dysfunction and the resulting vasoconstriction, which lead to pressure elevation in the pulmonary circulation (Boucherat et al., 2022). In the present study, MCT injection caused a robust elevation in RVSP, which was suppressed by treatment with magnesium nitrate. Additionally, magnesium nitrate halted pulmonary vascular remodeling, another characteristic feature of the MCT‐induced PH model (Boucherat et al., 2022), suggesting that it has beneficial pulmonary vascular effects against PH. However, the suppressive effects of magnesium nitrate on RV hypertrophy, a strong prognostic indicator of PH (Simpson et al., 2019), were not very strong. Various reports have shown data similar to ours, with weaker effects on RV hypertrophy than on pressure elevation and pulmonary vascular remodeling (Hou et al., 2023; Merklinger et al., 2005; Sadowska et al., 2020; Xie et al., 2012). Xie et al. showed that the long‐term administration of sildenafil (3 weeks from day 2 after MCT injection) suppressed pressure elevation, pulmonary vascular remodeling, and RV hypertrophy, whereas short‐term administration (2 weeks from day 23 after MCT injection) suppressed pressure elevation and pulmonary vascular remodeling, but not RV hypertrophy (Xie et al., 2012). Considering this evidence, it is possible that long‐term administration of magnesium nitrate could sufficiently inhibit RV hypertrophy. This is a topic for future research.

Lung edema leads to reduced lung compliance with subsequent hypoxemia; prolonged hypoxemia is an aggravating factor for PH and should be managed appropriately (Nathan et al., 2019). In this study, the administration of magnesium nitrate was not sufficient to inhibit MCT‐induced lung edema. In this PH model, lung edema has been shown to develop in the early phase before the onset of typical PH symptoms, such as RVSP elevation and pulmonary vascular remodeling (Veteskova et al., 2019), and was most likely already quite advanced at the time of initiating magnesium nitrate. In general, vasodilators are known to increase the risk of lung edema in certain types of PH (Humbert et al., 2022). To manage PH‐related lung edema, it may be better to use a combination of diuretics (Humbert et al., 2022).

Both NO_3_^−^ and Mg^2+^ levels in plasma were increased following the administration of magnesium nitrate in a dose‐dependent manner, which was consistent with the degree of improvement in PH symptoms. NO_3_^−^ is a possible source of NO in the body (Carlström et al., 2024), and NO has vasodilating and antiproliferative effects on vascular smooth muscle cells (Farah et al., 2018). Sustained NO supply to MCT‐injected rats resulted in lower pulmonary arterial pressure, no RV hypertrophy, and milder pulmonary vascular remodeling (Mathew et al., 1997). In studies on rat pulmonary arterial smooth muscle cells, NO was shown to induce apoptosis, a mechanism for the elimination of misguided proliferative cells via mitochondrial membrane depolarization (Krick et al., 2002), and to inhibit cell proliferation via p21^Waf1/Cip1^, a cyclin‐dependent kinase inhibitor (Zuckerbraun et al., 2010). On the other hand, Mg^2+^ has vasodilatory and anti‐inflammatory effects (de Baaij et al., 2015), as well as therapeutic effects against MCT‐induced PH (Chang et al., 2019; Mathew et al., 1988; Wang et al., 2021). One scientific report showed that Mg^2+^ inhibits pulmonary arterial smooth muscle cell proliferation and migration through the suppression of calcium signals for the nuclear factor of activated T cells 3 (NFATc3), an immune regulator (Wang et al., 2021). Unfortunately, it remains unclear whether the beneficial effects of magnesium nitrate on PH observed in the present study were due to NO_3_^−^, Mg^2+^, or both, and the underlying molecular mechanisms. This is a limitation of the present study.

Supplementation with potassium nitrate or sodium nitrate at 10 mM, the same concentration at which therapeutic effects were observed with magnesium nitrate, did not produce similar effects, confirming the superiority of magnesium nitrate in PH treatment. In line with our findings, Kuzenkov et al. reported that the protective effects of magnesium nitrate on neurological disorders are greater than those of potassium nitrate and sodium nitrate in rats with cerebral ischemia (Kuzenkov et al., 2013). However, it should be noted that the present study used a fixed dose and intervention period. In previous animal studies, potassium nitrate and sodium nitrate were shown to have therapeutic effects on PH (Baliga et al., 2012; Bubb et al., 2014; Ogoshi et al., 2018); therefore, it is possible for therapeutic effects to emerge following adjustments in dosage and intervention period.

There are several limitations to the findings in this study. Various studies have shown that calorie intake, circadian rhythm, and exercise volume affect disease severity of MCT‐induced PH (Ding et al., 2015; Paulin et al., 2020; Soares et al., 2019). We have not been able to verify whether magnesium nitrate affects food intake, sleep duration, and physical activity; future studies need to determine whether these factors contribute to the beneficial effects of magnesium nitrate on PH as confounding factors. Additionally, the MCT‐induced PH model might not fully represent the pathology in patients with PH. Therefore, more studies using cells and/or tissues derived from patients with PH are necessary to explore our findings in clinical situations.

In conclusion, our findings demonstrate that magnesium nitrate alleviates PH in rats by reducing RV pressure overload and inhibiting pulmonary vascular remodeling, providing significant information for its further development and investigation as an effective therapeutic agent for PH.

## AUTHOR CONTRIBUTIONS

MT: Conceived or designed study. MT, SA, AF, RA, and HY: Performed research and analyzed data. MT, KN, and MO: Wrote the paper.

## FUNDING INFORMATION

This study was partly supported by Grants‐in‐Aid for Scientific Research Program from the Japan Society for the Promotion of Science [Nos. 22K15299 and 25K14879 to MT].

## CONFLICT OF INTEREST STATEMENT

The authors have no relevant financial or nonfinancial interests to disclose.

## ETHICS STATEMENT

This study was performed in line with the Guide for the Care and Use of Laboratory Animals (8th Edition, 2011). Approval was granted by the Experimental Animal Committee at the Faculty of Pharmacy, Osaka Medical and Pharmaceutical University (Permit no: AP23‐021).

## Supporting information


Table S1.

Figure S1.

## Data Availability

The data supporting the findings of this study are available from the corresponding author upon reasonable request.

## References

[phy270416-bib-0001] Abidin, B. M. , Rios, F. J. , Montezano, A. C. , & Touyz, R. M. (2025). Transient receptor potential melastatin 7 cation channel, magnesium and cell metabolism in vascular health and disease. Acta Physiologica (Oxford, England), 241, e14282. 10.1111/apha.14282 39801180

[phy270416-bib-0002] Baliga, R. S. , Milsom, A. B. , Ghosh, S. M. , Trinder, S. L. , Macallister, R. J. , Ahluwalia, A. , & Hobbs, A. J. (2012). Dietary nitrate ameliorates pulmonary hypertension: Cytoprotective role for endothelial nitric oxide synthase and xanthine oxidoreductase. Circulation, 125, 2922–2932. 10.1161/CIRCULATIONAHA.112.100586 22572914 PMC3502837

[phy270416-bib-0003] Benza, R. L. , Grünig, E. , Sandner, P. , Stasch, J. P. , & Simonneau, G. (2024). The nitric oxide‐soluble guanylate cyclase‐cGMP pathway in pulmonary hypertension: From PDE5 to soluble guanylate cyclase. European Respiratory Review, 33, 230183. 10.1183/16000617.0183-2023 38508664 PMC10957071

[phy270416-bib-0004] Boucherat, O. , Agrawal, V. , Lawrie, A. , & Bonnet, S. (2022). The latest in animal models of pulmonary hypertension and right ventricular failure. Circulation Research, 130, 1466–1486. 10.1161/CIRCRESAHA.121.319971 35482834 PMC9060385

[phy270416-bib-0005] Bubb, K. J. , Trinder, S. L. , Baliga, R. S. , Patel, J. , Clapp, L. H. , MacAllister, R. J. , & Hobbs, A. J. (2014). Inhibition of phosphodiesterase 2 augments cGMP and cAMP signaling to ameliorate pulmonary hypertension. Circulation, 130, 496–507. 10.1161/CIRCULATIONAHA.114.009751 24899690 PMC4124037

[phy270416-bib-0006] Burke, M. M. , Bieger, D. , & Tabrizchi, R. (2011). Agonist‐induced periodic vasomotion in rat isolated pulmonary artery. Fundamental & Clinical Pharmacology, 25, 443–451. 10.1111/j.1472-8206.2010.00878.x 20880385

[phy270416-bib-0007] Carlström, M. , Weitzberg, E. , & Lundberg, J. O. (2024). Nitric oxide signaling and regulation in the cardiovascular system: Recent advances. Pharmacological Reviews, 76, 1038–1062. 10.1124/pharmrev.124.001060 38866562

[phy270416-bib-0008] Chang, C. Y. , Shih, H. J. , Huang, I. T. , Tsai, P. S. , Chen, K. Y. , & Huang, C. J. (2019). Magnesium sulfate mitigates the progression of monocrotaline pulmonary hypertension in rats. International Journal of Molecular Sciences, 20, 4622. 10.3390/ijms20184622 31540416 PMC6770589

[phy270416-bib-0009] Chaumais, M. C. , Lecerf, F. , Fattal, S. , Savale, L. , Günther, S. , Huertas, A. , Montani, D. , Perros, F. , Humbert, M. , & German‐Fattal, M. (2012). A study of magnesium deficiency in human and experimental pulmonary hypertension. Magnesium Research, 25, 21–27. 10.1684/mrh.2012.0301 22433438

[phy270416-bib-0010] de Baaij, J. H. , Hoenderop, J. G. , & Bindels, R. J. (2015). Magnesium in man: Implications for health and disease. Physiological Reviews, 95, 1–46. 10.1152/physrev.00012.2014 25540137

[phy270416-bib-0011] DeMartino, A. W. , Kim‐Shapiro, D. B. , Patel, R. P. , & Gladwin, M. T. (2019). Nitrite and nitrate chemical biology and signalling. British Journal of Pharmacology, 176, 228–245. 10.1111/bph.14484 30152056 PMC6295445

[phy270416-bib-0012] Ding, M. , Lei, J. , Qu, Y. , Zhang, H. , Xin, W. , Ma, F. , Liu, S. , Li, Z. , Jin, F. , & Fu, E. (2015). Calorie restriction attenuates monocrotaline‐induced pulmonary arterial hypertension in rats. Journal of Cardiovascular Pharmacology, 65, 562–570. 10.1097/FJC.0000000000000224 25636073 PMC4461391

[phy270416-bib-0013] Farah, C. , Michel, L. Y. M. , & Balligand, J. L. (2018). Nitric oxide signalling in cardiovascular health and disease. Nature Reviews Cardiology, 15, 292–316. 10.1038/nrcardio.2017.224 29388567

[phy270416-bib-0014] Fransen, P. , Van Hove, C. E. , Leloup, A. J. , Martinet, W. , De Meyer, G. R. , Lemmens, K. , Bult, H. , & Schrijvers, D. M. (2015). Dissecting out the complex Ca2+−mediated phenylephrine‐induced contractions of mouse aortic segments. PLoS One, 10, e0121634. 10.1371/journal.pone.0121634 25803863 PMC4372603

[phy270416-bib-0015] Fullerton, D. A. , Hahn, A. R. , Agrafojo, J. , Sheridan, B. C. , & McIntyre, R. C., Jr. (1996). Magnesium is essential in mechanisms of pulmonary vasomotor control. Journal of Surgical Research, 63, 93–97. 10.1006/jsre.1996.0229 8661179

[phy270416-bib-0016] Hou, C. , Xie, L. , Wang, T. , Zheng, J. , Zhao, Y. , Qiu, Q. , Yang, Y. , & Xiao, T. (2023). Comparative transcription profiling of mRNA and lncRNA in pulmonary arterial hypertension after C75 treatment. BMC Pulmonary Medicine, 23, 46. 10.1186/s12890-023-02334-6 36717804 PMC9887911

[phy270416-bib-0017] Humbert, M. , Kovacs, G. , Hoeper, M. M. , Badagliacca, R. , Berger, R. M. F. , Brida, M. , Carlsen, J. , Coats, A. J. S. , Escribano‐Subias, P. , Ferrari, P. , Ferreira, D. S. , Ghofrani, H. A. , Giannakoulas, G. , Kiely, D. G. , Mayer, E. , Meszaros, G. , Nagavci, B. , Olsson, K. M. , Pepke‐Zaba, J. , … ESC/ERS Scientific Document Group . (2022). ESC/ERS guidelines for the diagnosis and treatment of pulmonary hypertension. European Heart Journal, 43, 3618–3731. 10.1093/eurheartj/ehac237 36017548

[phy270416-bib-0018] Krick, S. , Platoshyn, O. , Sweeney, M. , McDaniel, S. S. , Zhang, S. , Rubin, L. J. , & Yuan, J. X. (2002). Nitric oxide induces apoptosis by activating K+ channels in pulmonary vascular smooth muscle cells. American Journal of Physiology—Heart and Circulatory Physiology, 282, H184–H193. 10.1152/ajpheart.2002.282.1.H184 11748062

[phy270416-bib-0019] Kuzenkov, V. S. , Krushinskii, A. L. , & Reutov, V. P. (2013). Effect of cation type and concentration of nitrates on neurological disorders during experimental cerebral ischemia. Bulletin of Experimental Biology and Medicine, 155, 748–751. 10.1007/s10517-013-2243-9 24288757

[phy270416-bib-0020] Mathew, R. , Gloster, E. S. , Altura, B. T. , & Altura, B. M. (1988). Magnesium aspartate hydrochloride attenuates monocrotaline‐induced pulmonary artery hypertension in rats. Clinical Science (London, England), 75, 661–667. 10.1042/cs0750661 2974771

[phy270416-bib-0021] Mathew, R. , Gloster, E. S. , Sundararajan, T. , Thompson, C. I. , Zeballos, G. A. , & Gewitz, M. H. (1997). Role of inhibition of nitric oxide production in monocrotaline‐induced pulmonary hypertension. Journal of Applied Physiology (1985), 82, 1493–1498. 10.1152/jappl.1997.82.5.1493 9134898

[phy270416-bib-0022] Merklinger, S. L. , Jones, P. L. , Martinez, E. C. , & Rabinovitch, M. (2005). Epidermal growth factor receptor blockade mediates smooth muscle cell apoptosis and improves survival in rats with pulmonary hypertension. Circulation, 112, 423–431. 10.1161/CIRCULATIONAHA.105.540542 16027270

[phy270416-bib-0023] Mocumbi, A. , Humbert, M. , Saxena, A. , Jing, Z. C. , Sliwa, K. , Thienemann, F. , Archer, S. L. , & Stewart, S. (2024). Pulmonary hypertension. Nature Reviews Disease Primers, 10, 1. 10.1038/s41572-023-00486-7 38177157

[phy270416-bib-0024] Nathan, S. D. , Barbera, J. A. , Gaine, S. P. , Harari, S. , Martinez, F. J. , Olschewski, H. , Olsson, K. M. , Peacock, A. J. , Pepke‐Zaba, J. , Provencher, S. , Weissmann, N. , & Seeger, W. (2019). Pulmonary hypertension in chronic lung disease and hypoxia. The European Respiratory Journal, 53, 1801914. 10.1183/13993003.01914-2018 30545980 PMC6351338

[phy270416-bib-0025] Nossaman, B. D. , Dabisch, P. A. , Liles, J. T. , Baber, S. R. , Champion, H. C. , Kaye, A. D. , Feng, C. J. , Anwar, M. , Bivalacqua, T. J. , Santiago, J. A. , De Witt, B. J. , & Kadowitz, P. J. (2004). Peroxynitrite does not impair pulmonary and systemic vascular responses. Journal of Applied Physiology (1985), 96, 455–462. 10.1152/japplphysiol.01159.2002 14715677

[phy270416-bib-0026] Ogoshi, T. , Tsutsui, M. , Kido, T. , Sakanashi, M. , Naito, K. , Oda, K. , Ishimoto, H. , Yamada, S. , Wang, K. Y. , Toyohira, Y. , Izumi, H. , Masuzaki, H. , Shimokawa, H. , Yanagihara, N. , Yatera, K. , & Mukae, H. (2018). Protective role of myelocytic nitric oxide synthases against hypoxic pulmonary hypertension in mice. American Journal of Respiratory and Critical Care Medicine, 198, 232–244. 10.1164/rccm.201709-1783OC 29480750

[phy270416-bib-0027] Paulin, R. , Lampron, M. C. , Vitry, G. , Grobs, Y. , Boucherat, O. , Provencher, S. , & Bonnet, S. (2020). Therapeutic potential of the nuclear receptor modulator SR9011 for treatment of pulmonary hypertension. American Journal of Respiratory and Critical Care Medicine, 201, A7672. 10.1164/ajrccm-conference.2020.201.1_MeetingAbstracts.A7672

[phy270416-bib-0028] Sadowska, O. , Baranowska‐Kuczko, M. , Gromotowicz‐Popławska, A. , Biernacki, M. , Kicman, A. , Malinowska, B. , Kasacka, I. , Krzyżewska, A. , & Kozłowska, H. (2020). Cannabidiol ameliorates monocrotaline‐induced pulmonary hypertension in rats. International Journal of Molecular Sciences, 21, 7077. 10.3390/ijms21197077 32992900 PMC7582795

[phy270416-bib-0029] Simpson, C. E. , Damico, R. L. , Kolb, T. M. , Mathai, S. C. , Khair, R. M. , Sato, T. , Bourji, K. , Tedford, R. J. , Zimmerman, S. L. , & Hassoun, P. M. (2019). Ventricular mass as a prognostic imaging biomarker in incident pulmonary arterial hypertension. The European Respiratory Journal, 53, 1802067. 10.1183/13993003.02067-2018 30705128 PMC7263169

[phy270416-bib-0030] Soares, L. L. , Drummond, F. R. , Rezende, L. M. T. , Lopes Dantas Costa, A. J. , Leal, T. F. , Fidelis, M. R. , Neves, M. M. , Prímola‐Gomes, T. N. , Carneiro‐Junior, M. A. , Carlo Reis, E. C. , & Natali, A. J. (2019). Voluntary running counteracts right ventricular adverse remodeling and myocyte contraction impairment in pulmonary arterial hypertension model. Life Sciences, 238, 116974. 10.1016/j.lfs.2019.116974 31639399

[phy270416-bib-0031] Suzuki, T. , Karaki, H. , & Urakawa, N. (1981). Inhibition of contraction by swelling of vascular smooth muscle in high KCl, low Na solution. Archives Internationales de Pharmacodynamie et de Thérapie, 250, 195–203. 7271384

[phy270416-bib-0032] Tawa, M. , Nagano, J. , Kitama, J. , Abe, S. , Fujita, A. , Nakagawa, K. , & Ohkita, M. (2022). Ameliorative effects of beetroot juice supplementation on monocrotaline‐induced pulmonary hypertension in rats. Future Pharmacology, 2, 547–557. 10.3390/futurepharmacol2040033

[phy270416-bib-0033] Tawa, M. , Nagata, R. , Sumi, Y. , Nakagawa, K. , Sawano, T. , Ohkita, M. , & Matsumura, Y. (2021). Preventive effects of nitrate‐rich beetroot juice supplementation on monocrotaline‐induced pulmonary hypertension in rats. PLoS One, 16, e0249816. 10.1371/journal.pone.0249816 33831045 PMC8031446

[phy270416-bib-0034] Tolsa, J. F. , Gao, Y. , & Raj, J. U. (1999). Developmental change in magnesium sulfate‐induced relaxation of rabbit pulmonary arteries. Journal of Applied Physiology (1985), 87, 1589–1594. 10.1152/jappl.1999.87.5.1589 10562595

[phy270416-bib-0035] Veteskova, J. , Kmecova, Z. , Malikova, E. , Doka, G. , Radik, M. , Vavrinec, P. , Krenek, P. , & Klimas, J. (2019). Opposite alterations of endothelin‐1 in lung and pulmonary artery mirror gene expression of bone morphogenetic protein receptor 2 in experimental pulmonary hypertension. Experimental Lung Research, 45, 30–41. 10.1080/01902148.2019.1605426 31012341

[phy270416-bib-0036] Villamor, E. , Pérez‐Vizcaíno, F. , Ruiz, T. , Tamargo, J. , & Moro, M. (1996). In vitro effects of magnesium sulfate in isolated intrapulmonary and mesenteric arteries of piglets. Pediatric Research, 39, 1107–1112. 10.1203/00006450-199606000-00029 8725278

[phy270416-bib-0037] Vilskersts, R. , Kuka, J. , Liepinsh, E. , Cirule, H. , Gulbe, A. , Kalvinsh, I. , & Dambrova, M. (2014). Magnesium nitrate attenuates blood pressure rise in SHR rats. Magnesium Research, 27, 16–24. 10.1684/mrh.2014.0358 24827813

[phy270416-bib-0038] Wang, D. , Zhu, Z. L. , Lin, D. C. , Zheng, S. Y. , Chuang, K. H. , Gui, L. X. , Yao, R. H. , Zhu, W. J. , Sham, J. S. K. , & Lin, M. J. (2021). Magnesium supplementation attenuates pulmonary hypertension via regulation of magnesium transporters. Hypertension, 77, 617–631. 10.1161/HYPERTENSIONAHA.120.14909 33356397

[phy270416-bib-0039] Xie, Y. P. , Chen, B. , Sanders, P. , Guo, A. , Li, Y. , Zimmerman, K. , Wang, L. C. , Weiss, R. M. , Grumbach, I. M. , Anderson, M. E. , & Song, L. S. (2012). Sildenafil prevents and reverses transverse‐tubule remodeling and Ca(2+) handling dysfunction in right ventricle failure induced by pulmonary artery hypertension. Hypertension, 59, 355–362. 10.1161/HYPERTENSIONAHA.111.180968 22203744 PMC3266850

[phy270416-bib-0040] Xing, J. , Wang, M. , Hong, J. , Gao, Y. , Liu, Y. , Gu, H. , Dong, J. , & Li, L. (2019). TRPM7 channel inhibition exacerbates pulmonary arterial hypertension through MEK/ERK pathway. Aging (Albany NY), 11, 4050–4065. 10.18632/aging.102036 31219801 PMC6629001

[phy270416-bib-0041] Zhuang, X. L. , Zhu, Z. L. , Huang, Q. H. , Yan, F. R. , Zheng, S. Y. , Lai, S. M. , Jiao, H. X. , & Lin, M. J. (2022). High magnesium mitigates the vasoconstriction mediated by different types of calcium influx from monocrotaline‐induced pulmonary hypertensive rats. Experimental Physiology, 107, 359–373. 10.1113/EP090029 35193162

[phy270416-bib-0042] Zuckerbraun, B. S. , Shiva, S. , Ifedigbo, E. , Mathier, M. A. , Mollen, K. P. , Rao, J. , Bauer, P. M. , Choi, J. J. , Curtis, E. , Choi, A. M. , & Gladwin, M. T. (2010). Nitrite potently inhibits hypoxic and inflammatory pulmonary arterial hypertension and smooth muscle proliferation via xanthine oxidoreductase‐dependent nitric oxide generation. Circulation, 121, 98–109. 10.1161/CIRCULATIONAHA.109.891077 20026772

