# Supplementary material for: Beneficial effects of magnesium nitrate on disease severity in male rats with monocrotaline‐induced pulmonary hypertension
Source: Physiol Rep. 2025 Jun 17;13(12):e70416. doi: 10.14814/phy2.70416 (PMC12172563; doi:10.14814/phy2.70416)
Supplement: Supplementary file 1 — Table S1. [file PHY2-13-e70416-s001.docx]

**TABLE S1.**

Various parameters in saline- and MCT-injected rats

|  | Control (*n* = 3) | MCT (*n* = 6) | PN (*n* = 7) | SN (*n* = 7) |
| --- | --- | --- | --- | --- |
| BW (g) | 434.7 ± 23.0^**^ | 357.7 ± 22.5 | 342.9 ± 32.2 | 367.7 ± 24.7 |
| RVW/BW (g/kg) | 0.48 ± 0.02^**^ | 1.09 ± 0.17 | 1.06 ± 0.19 | 0.94 ± 0.23 |
| LW/BW (mg/g) | 3.37 ± 0.20^**^ | 6.88 ± 0.99 | 6.84 ± 1.17 | 6.86 ± 1.52 |
| HR (bpm) | 374.8 ± 18.2 | 367.9 ± 30.9 | 384.5 ± 44.0 | 407.0 ± 38.9 |
| MAP (mmHg) | 92.5 ± 8.5 | 79.3 ± 13.0 | 84.9 ± 10.2 | 91.9 ± 18.7 |

Data are the mean ± SD of 3–7 experiments. ***p* < 0.01, compared with the MCT group. Statistical analysis was performed using one-way ANOVA and the Holm–Sidak post-hoc test. BW, body weight; HR, heart rate; LW, lung weight; MAP, mean arterial pressure; MCT, monocrotaline; PN, potassium nitrate; RVW, right ventricular weight; SN, sodium nitrate.
