# Supplementary material for: Beneficial effects of magnesium nitrate on disease severity in male rats with monocrotaline‐induced pulmonary hypertension
Source: Physiol Rep. 2025 Jun 17;13(12):e70416. doi: 10.14814/phy2.70416 (PMC12172563; doi:10.14814/phy2.70416)

**FIGURE S1.**

PH symptoms in saline- and MCT-injected rats; (a) RVSP; (b) medial thickness; (c) RVW to (LV + S)W ratio; (d) plasma NO_3_^-^ levels. Each point represents an individual value; each column and bar represent the mean ± SD of 3–7 experiments. Numbers above the line marks represent calculated *p* value. Statistical analysis was performed using one-way ANOVA and the Holm–Sidak post-hoc test. (LV + S)W, left ventricular plus septum weight; MCT, monocrotaline; NO_3_^-^, nitrate; PH, pulmonary hypertension; PN, potassium nitrate; RVSP, right ventricular systolic pressure; RVW, right ventricular weight; SN, sodium nitrate.


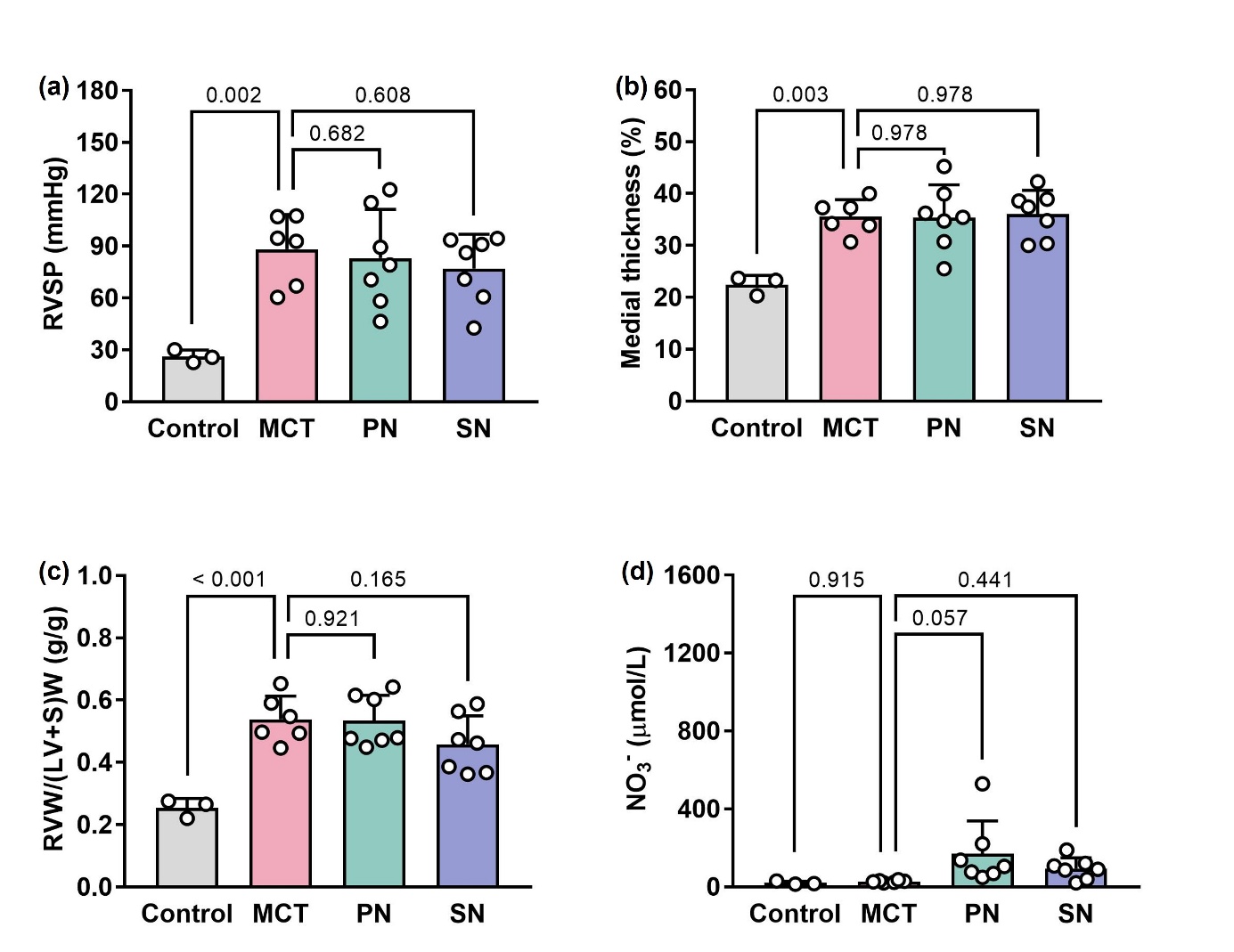

Supplement: Supplementary file 2 — Figure S1. [file PHY2-13-e70416-s002.docx]
